# Structural Covariance of Sensory Networks, the Cerebellum, and Amygdala in Autism Spectrum Disorder

**DOI:** 10.3389/fneur.2017.00615

**Published:** 2017-11-27

**Authors:** Garrett J. Cardon, Susan Hepburn, Donald C. Rojas

**Affiliations:** ^1^Department of Psychology, Colorado State University, Fort Collins, CO, United States; ^2^Department of Human Development and Family Studies, Colorado State University, Fort Collins, CO, United States

**Keywords:** structural covariation, autism spectrum disorder, sensory dysfunction, cerebellum, amygdala

## Abstract

Sensory dysfunction is a core symptom of autism spectrum disorder (ASD), and abnormalities with sensory responsivity and processing can be extremely debilitating to ASD patients and their families. However, relatively little is known about the underlying neuroanatomical and neurophysiological factors that lead to sensory abnormalities in ASD. Investigation into these aspects of ASD could lead to significant advancements in our general knowledge about ASD, as well as provide targets for treatment and inform diagnostic procedures. Thus, the current study aimed to measure the covariation of volumes of brain structures (i.e., structural magnetic resonance imaging) that may be involved in abnormal sensory processing, in order to infer connectivity of these brain regions. Specifically, we quantified the structural covariation of sensory-related cerebral cortical structures, in addition to the cerebellum and amygdala by computing partial correlations between the structural volumes of these structures. These analyses were performed in participants with ASD (*n* = 36), as well as typically developing peers (*n* = 32). Results showed decreased structural covariation between sensory-related cortical structures, especially between the left and right cerebral hemispheres, in participants with ASD. In contrast, these same participants presented with increased structural covariation of structures in the right cerebral hemisphere. Additionally, sensory-related cerebral structures exhibited decreased structural covariation with functionally identified cerebellar networks. Also, the left amygdala showed significantly increased structural covariation with cerebral structures related to visual processing. Taken together, these results may suggest several patterns of altered connectivity both within and between cerebral cortices and other brain structures that may be related to sensory processing.

## Introduction

Diagnostic criteria for autism spectrum disorder (ASD) underwent revision in 2013 [DSM 5 (1)]. One major change to the criteria is that sensory dysfunction was added as a symptom area able to meet criterion B.4 (restricted and repetitive behaviors, interests, or activities), highlighting its importance as a core feature of ASD. Despite this recent recognition, as well as estimates of the prevalence of sensory problems in ASD exceeding 90% (2–5), sensory dysfunction in ASD is poorly understood. This gap is especially notable with respect to the neurobiological underpinnings of sensory dysfunction (6). Gaining knowledge about sensory dysfunction in ASD is needed in order to devise ways to ameliorate their debilitating effects on patients and their families. Neuroimaging techniques, such as magnetic resonance imaging (MRI), can provide opportunities to better describe neurophysiologic correlates of sensory dysfunction in humans with ASD. The current study aimed to investigate anatomical relationships between cortical and subcortical structures involved in sensory processing as well as the cerebellum and amygdala as an initial step toward examining the neurobiological underpinnings of sensory dysfunction in ASD.

Changes in brain connectivity are a theme emerging across several theories of ASD [i.e., Weak Central Coherence, Predictive Coding, Reduced Sensory Precision, Temporal Binding Deficit, and Excitatory/Inhibitory Imbalance; e.g., (7–16)]. These perspectives suggest that phenotypes associated with ASD are subserved by deficits in distributed neurological networks, rather than single portions of the brain. Indeed, the available literature on the neural bases of sensory dysfunction in ASD suggests that unisensory subcortical and cortical processing, though involved, is likely not the only process contributing to abnormal sensory responsivity (17). Rather, evidence points to other, supra-modal, brain structures that may also be involved in sensory dysfunction. For example, one of the most often reported structural abnormalities in ASD is found in the cerebellum (18–24). Both Purkinje cell loss (18) and decreased cerebellar gray matter volumes (19–22, 25, 26) have consistently been shown in ASD relative to control subjects. While the cerebellum is typically thought of in terms of its role in motor function (27), the cerebellum also plays a role in both multisensory integration (24, 28) and prediction of sensory input (29). Its multisensory integration function is supported by the fact that the cerebellum receives projections from all sensory modalities and the areas of the cerebellum to which these sensory systems project often overlap (24, 30). For instance, self-motion requires integration of vestibular, visual, proprioceptive, and somatosensory information. Specifically, vestibular and proprioceptive information is combined with multiple sensory modalitys’ information in the cerebellum to generate representations about head and body position, translation, and tilt, and heading direction (17). A cerebellar deficiency would therefore negatively affect responses to sensory stimuli, regardless of the modality, by hampering multisensory integration, and both the ability to anticipate sensory events and prepare appropriate response to the same.

Additionally, overstimulation perceived as threatening could be related to enhanced fear responses in ASD, which would likely be mediated by non-sensory-specific brain regions (31–33). Following this line of reasoning, the amygdala could be postulated as involved in the abnormal sensory responsivity in ASD, given its classic role in fear processing, its connections to sensory systems, and oft reported abnormalities in ASD (6, 34–36). For example, it has been shown that the degree to which the amygdala is stimulated during a sensory event predicts the extent to which that sensory experience is deemed unpleasant or threatening (34). Thus, it is plausible to hypothesize that in addition to sensory-specific cortical regions, other brain areas such as the cerebellum and amygdala may be critical to sensory dysfunction and reactivity in ASD.

Establishing the notion of distributed network involvement in sensory dysfunction necessitates measures of neural connectivity and co-activation. For instance, studies involving functional connectivity (i.e., covariation of the BOLD response between regions of interest in the brain) have shown significant differences between participants with ASD and those who were typically developing [TD (37–40)]. One of the most common functional connectivity findings reveals that local, within-region connectivity is enhanced, while long-range connections appear weakened in ASD, relative to controls, especially in the default mode network [DMN (11–13, 15, 29, 37, 41–44)]. In addition to functional connectivity, some previous reports have also shown significant differences between those with ASD and TD peers in the structural features of their brains [see Pua et al. (45) for a review]. Furthermore, the covariation of structural attributes of distinct brain regions (i.e., volume, thickness, surface area) has recently been used as a measure of connectivity (46). This type of analysis has been termed morphological connectivity, although to avoid conflating the term with more direct functional and structural metrics of connectivity, we prefer the term covariation. The assumption of morphological covariation is that regions of the brain that are connected and co-active also tend to covary in their structural characteristics. These structural relationships may be mediated by common experience-dependent plasticity or mutually trophic influences (46). A number of studies have found significant results using structural/morphological covariation as a measure of related brain regions in ASD vs. TD controls (47, 48). In fact, recently, several investigations have reported findings that support the use of morphological covariation as a means to distinguish participants with ASD from TD subjects (48). However, no study of morphological covariation in ASD has focused specifically on neural centers related to sensory processing and dysfunction, to our knowledge. Thus, the current investigation aimed to evaluate the morphological covariation between cortical regions known to be associated with sensory function, such as the temporal and occipital cortices and post-central gyrus, as well as supramodal brain areas that may be instrumental in sensory processing and dysfunction in ASD, including the cerebellum, amygdala, and language-related areas (e.g., supramarginal gyrus and caudal medial prefrontal cortex). We hypothesized that local morphological covariation would be enhanced, while long-range covariation would be decreased in individuals with ASD, compared to controls. Additionally, we predicted that structural correlations between the cerebellum and sensory cortices would be weaker in ASD compared with controls. Finally, we hypothesized that sensory cortices would exhibit stronger covariance to the amygdala in those with ASD relative to matched controls.

## Materials and Methods

### Participants

Participants for the current study consisted of two groups of male participants: (1) individuals with ASD (*n* = 36; mean age = 18.24 years, SD = 9.9); (2) TD individuals (*n* = 32; mean age = 18.9 years, SD = 12.28). Ages of these groups did not differ significantly [*t*(66) = −0.26, *p* = 0.80]. ASD subjects were diagnosed using a convergence of meeting criteria on (1) the Autism Diagnostic Observation Schedule—Generic [ADOS-G (49)], (2) the Autism Diagnostic Interview Revised (ADI^TM^-R) (50) or Social Communication Questionnaire [SCQ (51)], and (3) confirmation of the diagnosis by a clinical psychologist with expertise in ASD using a DSM-IV checklist during a clinical interview. A second psychologist reviewed case diagnostic data and independently formulated a DSM-IV diagnosis. The ASD diagnosis therefore employed DSM-IV criteria and included Autistic Disorder (*n* = 19), Asperger’s Syndrome (*n* = 15), and PDD-NOS (*n* = 2). Agreement on ASD vs. not ASD was 100% and agreement on specific ASD sub-diagnosis was 88%. Severity of ASD symptoms was measured using the Social Responsiveness Scale, Second Edition [(52); SRS-2], a 65-item questionnaire that measures several aspects of social functioning that accurately distinguish ASD from other psychiatric disorders. As a group, those with ASD presented with a mean score of 96.79 (SD = 25.5), which is in the severe symptom range and is highly indicative of clinical diagnoses of ASD. Non-verbal IQ (Wechsler Abbreviated Scale of Intelligence) scores were available in a subset of individuals from both the ASD (*n* = 32) and TD (*n* = 27) groups and were as follows: ASD—mean IQ = 111.19, SD = 14.95; TD—mean IQ = 116.26, SD = 12.46. The ASD and TD groups did not differ significantly in non-verbal IQ (*t* = −1.39; *p* = 0.25). Individuals were excluded from the study if they: (1) had a known genetic etiology of ASD (e.g., Fragile X Syndrome, Tuberous Sclerosis, 15q syndrome, etc.); (2) had a full-scale IQ below 70; (3) had a history of seizure disorder; or (4) had a history of brain injury, stroke, or other neurological disorder. All participants were recruited in accordance with human subjects protection policies of the Colorado Multiple Institutional Review Board (COMIRB) of the University of Colorado Denver Anschutz Medical Campus, where MRI scanning took place.

### MRI Acquisition

The T1-weighted structural MRI scan data used in the current study were obtained from participants in an NIH-funded study to DCR concerning magnetoencephalographic brain activity in ASD, and as such were not specifically acquired to answer questions of sensory processing abnormality. The data were acquired using a 3.0 T GE Signa HDx long-bore MR scanner (General Electric Hardware, WI, USA) together with a GE 8-channel phased-array head coil. In order to minimize participant motion and to improve compliance, subjects were allowed to watch and listen to a movie through MR compatible goggles and head phones (Resonance Technology, Inc., Northridge, CA, USA). For tissue segmentation, a T1-weighted sequence was acquired using a 3D inversion recovery, fast, spoiled gradient echo (IR-SPGR) technique (matrix = 256^2^, FOV 22 cm, TR/TE/TI = 10/3/450 ms, NEX = 1). MR acquisitions resulted in 138, 1.2 mm thick axial slices with an in-plane resolution of 0.86 mm^2^.

### Structural Covariation and Statistical Analysis

Volumetric measurements for the sensory and supramodal structures of interest were calculated using the Freesurfer 5.3 image analysis suite (http://surfer.nmr.mgh.harvard.edu/). Freesurfer has been used widely to perform automatic volumetric calculation and has shown good test–retest reliability across manufacturers of MR scanners, as well as field strengths (53, 54). The details behind the extraction of volumetric data have been well described in previous publications (53–66). In general, cortical surface reconstruction was performed *via* the following preprocessing steps: intensity normalization, skull stripping, pial surface generation, and use of triangular tessellation to generate a white/gray matter boundary [as in Tanabe et al. (67)]. Manual edits were made to volumes and surfaces as needed to correct issues remaining after automated processes.

Fifty-four anatomically distinct brain regions were included in the volumetric analysis (68). In general, the structures consisted of subdivisions of the frontal, temporal, occipital, and parietal cortices, as well as the amygdala. In addition, information regarding the volumes of the cerebellar hemispheres and seven functionally distinct cerebellar networks was extracted from the structural MRI scan by applying a cerebellar network template (69, 70) to participants’ scans. Once obtained, volumetric data were entered into the SPSS software package and Matlab for statistical analysis [(71); version 23; The Mathworks, version 2014b with Statistical Toolbox]. In order to compare volumetric data between groups, a general linear model was formed with structural volumes as the dependent variables, diagnosis as the independent variable, and intracranial volume as a covariate. Findings from this test were subjected to multiple comparisons adjustment using a false discovery rate (FDR) adjustment procedure [*q* = 0.1 (72)]. Following this between groups analysis, partial (i.e., removing the effects of intracranial volume and age) correlation coefficients were permuted (randomized labeling exchanges between subjects) between all structure pairs (10,000 permutations for each pair). Non-parametric *p*-values were then obtained by taking the number of permuted partial correlation coefficients higher than the actual partial correlation for each pair by the total number of permutations (absolute values of the coefficients were used in this procedure to yield two-tailed results). These non-parametric partial correlation *p*-values were then subjected to multiple comparisons adjustment by FDR.

Finally, between group differences in the correlation data were assessed for all structure pairings as follows: (1) a Fisher’s r to z transform was computed to obtain z-scores, (2) z-scores were compared according to the formula from Cohen and Cohen (73), and (3) *p*-values were obtained *via* permutations of the group membership (10,000 permutations each with randomized group label exchanges).

## Results

### Between Group Structural Volumetric Comparisons

All volume statistics included intracranial volume as covariates to account for general variability of brain size. Between groups comparison of absolute structural volumes revealed that several structures differed significantly between the ASD and TD groups. For example, the left and right transverse temporal volumes were significantly larger in the ASD vs. the TD group [left: *F*(1, 54) = 13.73; *q* = 0.00; right: *F*(1, 54) = 10.99; *q* = 0.03]. On the other hand, the right banks of the superior temporal sulcus was significantly smaller in the ASD group [*F*(1, 54) = 10.91; *q* = 0.03]. Also, the nucleus accumbens from both the left and right hemispheres were both significantly smaller overall in the participants with ASD [left: *F*(1, 54) = 7.77; *q* = 0.08; right: *F*(1, 54) = 16.12; *q* = 0.00]. Significance, mean, and SD volume values can be seen in Table S1 in Supplementary Material. Significant differences in these particular brain regions, which are highly associated with sensory processing, may be related to abnormalities in sensory function in the ASD group.

### Sensory Cortical Structural Covariance

**Figure 1:**
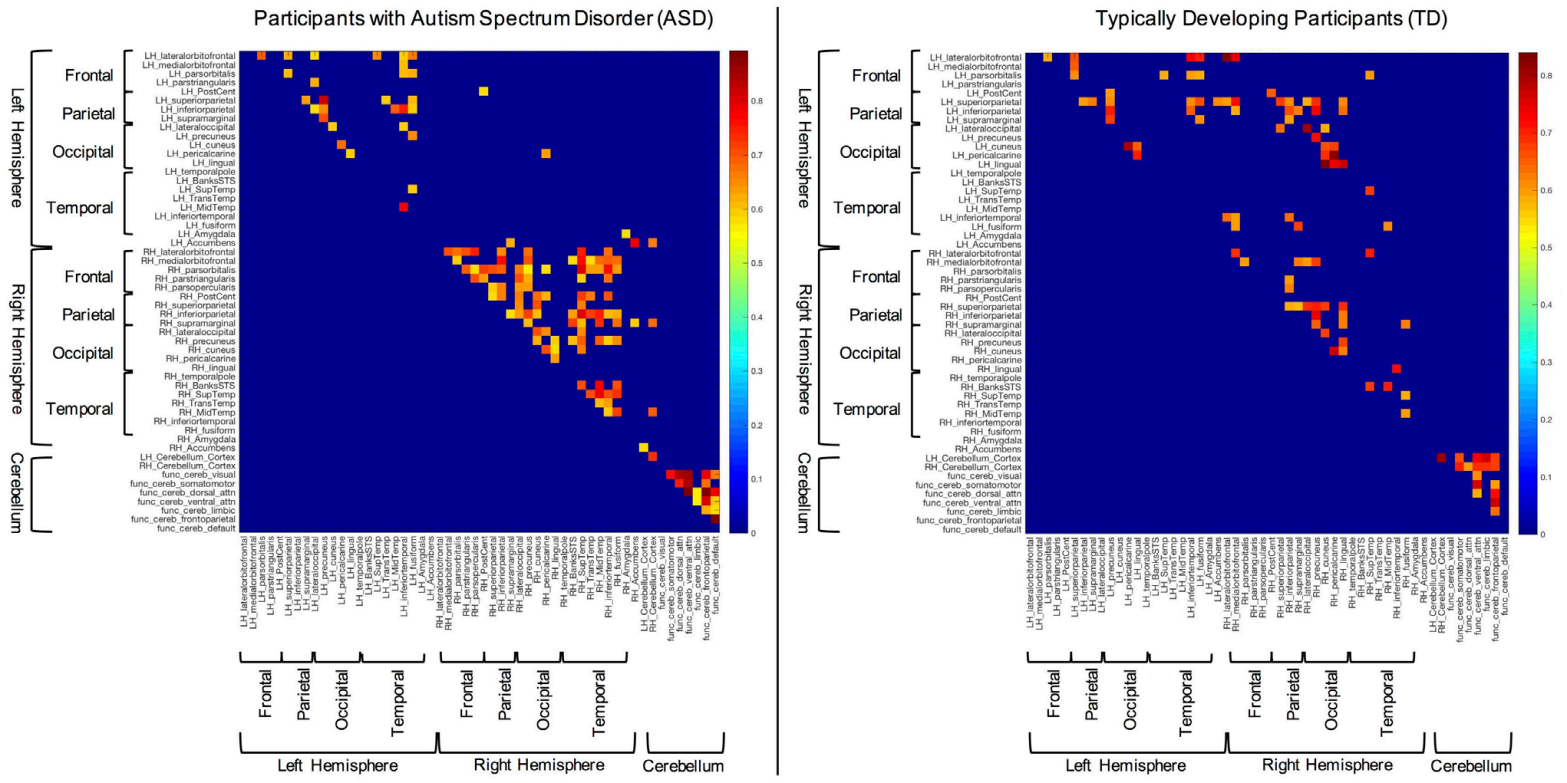
Significant structural correlations in the autism spectrum disorder and typically developing (TD) groups (following multiple comparisons correction). Hemispheres, lobes, and cerebellum are marked with brackets for convenience.

The structural (i.e., partial) correlations that remained significant following multiple comparisons correction for both the ASD and TD groups can be seen in Figure 1. These data reveal several notable findings with respect to the hypotheses of the current study. For instance, subjects with ASD present with few significant correlations between the right and left hemispheres compared to TD participants (i.e., inter-hemispheric covariation). In contrast, significant within hemisphere (i.e., intra-hemispheric covariation) structural correlations seem more abundant than inter-hemispheric correlations in the ASD group, especially within the right hemisphere.

### Between Groups Comparison of Structural Covariation

**Figure 2:**
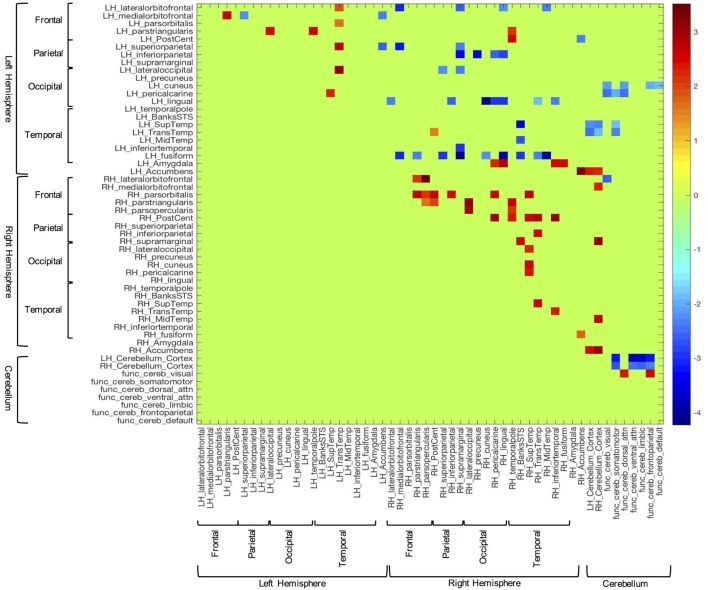
Z-scores of structural volume correlations masked by the indices of significantly different between group correlations (corrected for multiple comparisons). Blue cells represent z-scores associated with stronger correlations in the typically developing group, while red cells correspond to stronger correlations from the autism spectrum disorder group.

In order to examine between group differences in structural covariation, we plotted the z-scores of the correlations that were significantly different between groups (i.e., Fisher’s z transform). These data can be seen in Figure 2. In this figure, blue cells are indicative of correlations that were stronger in the TD group, while red cells show correlations that were stronger in the ASD group. Consistent with the above results, overall, the TD group showed significantly stronger inter-hemispheric and cerebellar cortex–functional cerebellar network correlations than the ASD group. On the other hand, the ASD participants presented with significantly more intra-hemispheric correlations.

Also germane to the current hypotheses, the functional networks of the cerebellum (69, 70) showed fewer correlations with sensory-related structures in the ASD group, vs. controls. Notably, the control group showed significant covariation between the cerebellar network associated with somatomotor function and the left pericalcarine (*z* = −2.04; *p* = 0.03), superior temporal (*z* = −1.90; *p* = 0.03), and transtemporal cortices (*z* = −2.34; *p* = 0.03). Additionally, this group exhibited several significant correlations between the dorsal attention functional cerebellar networks and visual cortices [i.e., left cuneus (*z* = −2.15; *p* = 0.04) and pericalcarine cortex (*z* = −2.42; *p* = 0.04)]. In contrast, the ASD group did not show any significant correlations in the above areas.

In the between groups comparison, the covariation between the amygdala and sensory-related structures showed the opposite pattern compared to the cerebellum. That is, the TD group showed no significantly stronger correlations with the amygdala, while those with ASD showed several correlations of note. For instance, the volume of the left amygdala was more strongly correlated with several cortical areas associated with visual processing, including some highly implicated in facial processing [i.e., right pericalcarine (*z* = 2.29; *p* = 0.05), lingual (*z* = 2.91; *p* = 0.03), inferior temporal (*z* = 2.45; *p* = 0.04), and fusiform cortices (*z* = 2.48; *p* = 0.04)].

## Discussion

The results of the current study suggest that individuals with ASD present with altered structural volumes and covariance in brain regions that may be associated with sensory processing and reactivity, prediction, and emotion. The following findings support these notions: (i) participants with ASD exhibited larger right and left transverse temporal gyrus volumes, while these same subjects presented with smaller overall volumes of the right banks of the STS, and left and right nucleus accumbens, relative to TD participants (see Between Group Structural Volumetric Comparisons); (ii) increased covariation was seen between structural volumes of sensory-related cortices within the right and, to a lesser degree, left hemispheres of persons with ASD vs. TD subjects. In contrast, ASD participants showed decreased covariation of structural volumes of sensory-related cortices between the right and left cerebral hemispheres, compared to the TD group (Figures 1 and 2); (iii) overall, the ASD group showed differences in structural covariation between the cerebellum and sensory-related cerebrum, in contrast to the TD group (Figure 2); (iv) the ASD group presented with a greater number of significant amygdala–sensory cortical correlations than TD peers, especially in the right hemisphere. Furthermore, the amygdalae of ASD participants showed significantly increased average structural volumetric correlations to the right occipital and temporal cortices (Figure 2).

### Inter- vs. Intra-Hemispheric Correlations of Sensory Cortical Structures

Various studies, using both structural and functional techniques, have demonstrated altered cortical network characteristics in ASD. Probably the most common finding among these studies concerns local hyperconnectivity, with hypo-connectivity of long-range circuits (12, 13, 15, 38, 74–78). This type of result has been shown, for example, in the DMN, in which increased connectivity was seen in local network nodes, while longer-range connections running in an anterior–posterior orientation were compromised (38, 76). Additionally, decreased inter-hemispheric and cerebellar-cerebral (i.e., long-range) resting sate functional connectivity (75, 79), as well as increased right hemisphere connectivity [i.e., shorter-range (76, 80)] have been reported in ASD. Such a connectivity pattern might leave specialized information processing units isolated from other brain regions, because of the lack of global connectivity (7, 8). The results of the current study, indicating that local correlation within both cortical hemispheres was increased, coupled with decreased inter-hemispheric correlations, are consistent with the above notions. This structural covariance pattern may reflect hyperconnectivity of specialized local sensory networks and isolation of the same due to deficient inter-hemispheric connections. Within local networks, this type of finding may be related to behavioral hyper-arousal and hyper-focus on certain sensory inputs in ASD (81–83). Also, both short- and long-range sensory covariance results could be associated with symptoms of weak central coherence—another commonly reported theory in ASD (7).

One area of sensory processing that has been highly implicated in ASD is multisensory integration. That is, numerous investigators have argued that individuals with ASD have difficulty processing various streams of simultaneous sensory input [see Marco et al. (4), for a review]. Indeed, neurophysiologic findings have corroborated these arguments. For instance, subjects with ASD have been shown to have deficits in processing illusions, such as the McGurk effect, which rely on integration of multiple sensory inputs (84). Findings from the current study may elucidate neurobiological underpinnings of these deficiencies in multisensory integration. For instance, the clear lack of volumetric correlation between the cerebral hemispheres may suggest that sensory cortices are not communicating with each other normally, assuming that such communication results in mutually trophic influence. Such a lack of neural connectivity could contribute to disordered multisensory integration. That is, white matter abnormalities can lead to deficiencies in processing the precise timing of action potentials, which is a prerequisite for accurate sensory processing and multisensory integration (85). One previous study showed significant correlations between behavioral measures of sensory processing and multisensory integration and white matter abnormalities, including those in the mid posterior region of the corpus callosum, in children with sensory processing disorder (85).

### Cerebellum–Cortex Correlations

The difference in significant cerebellar–cortex correlations seen in the present study between the ASD and TD groups may be indicative of altered connectivity between these brain regions in the former. Decreased covariation between cerebellum and sensory cortices could be related to abnormal sensory reactivity in ASD in a number of ways. For instance, Courchesne and Allen (29) have theorized that the cerebellum monitors sensory inputs and uses them to create predictions of future events, based on past experience, and then prepares the organism to respond to these stimuli. Disruptions in this predictive ability tend to lead to deficiencies in predicting sensory events and adaptive responses to the same. Differences between predicted sensory occurrences and actual sensory input (i.e., prediction errors) could lead to sensory stimulation being perceived as strange, unpleasant, surprising, and/or overwhelming (16, 29, 86, 87). Given the cellular, structural, and functional connectivity-based abnormalities that have been reported in ASD (18, 19, 21, 79), the sensory inputs to the cerebellum, and its role in prediction, one might reason that cerebellar deficiency might plausibly be related to sensory dysfunction in ASD. Additionally, the cerebellum may play an important role in multisensory integration (24, 28), as it typically receives and sends projections from and to sensory cortices. For example, many of these projections come from the superior colliculus (SC), where, especially, auditory and visual sensory inputs are combined to form a multisensory representation of various aspects of our environment. Once information from the SC is sent to the cerebellum (particularly the vermis, lobules VI and VII), it is modulated—either enhanced or depressed—and sent back to the SC, where it is sent both to the cortex and subcortical areas. Abnormal integration or modulation of multisensory information could lead to inaccuracies or confusion in their interpretation and the responses to the same (24). Thus, it is plausible that the connectivity between the cerebellum and sensory cortices contributes a great deal to sensory processing, and that abnormalities in these connections could lead to sensory malfunction.

### Amygdala–Cortex Correlation

It is reasonable to believe that sensory input perceived as threatening (i.e., hyper-reactivity) would likely be mediated, at least in part, by the amygdala (33). Zald (34) argued that the degree to which the amygdala is stimulated during a sensory event predicted the extent to which that sensory experience was considered unpleasant or threatening. Abnormalities of the amygdala have often been reported in ASD. For example, in the VPA rat model of ASD, affected rats were shown to have overactive amygdalae (32), which lead to hyper-reactivity, decreased inhibition, and boosted synaptic plasticity. These factors were correlated with heightened behavioral fear responsivity in these animals. Consistent with animal studies, recent studies performed in humans also found ASD subjects’ amygdalae and primary auditory and somatosensory cortices to be overreactive during mildly aversive sensory stimuli, when compared to controls (6). This and a related study also both showed that the BOLD responses of ASD amygdalae were positively correlated with behavioral measures of sensory over-reactivity in these individuals (6, 36). The current study showed significantly increased structural covariance between the amygdalae and right occipital and temporal cortices, which may be suggestive of hyperconnectivity similar to that reported in the aforementioned investigations. Most of the areas that showed a significantly higher degree of correlation with the amygdalae of those with ASD, vs. TD participants, seem important for facial and human body processing—e.g., inferior temporal, fusiform, and lingual gyri. Hyperconnectivity between amygdalae and such areas may contribute to the social deficits which are common in ASD [see Schultz (88), for a review].

### Hemispheric Asymmetry

Hemispheric asymmetries are commonly reported in ASD, especially as they relate to cortical regions associated with language processing. Some language-related regions such as planum temporale exhibit leftward asymmetry (89, 90). In ASD, specific asymmetry findings in such structures are mixed, but consistently show asymmetry changes (91–93). For example, studies by Rojas et al. (91, 92) showed reduced planum temporale asymmetry, with the left planum temporale smaller in ASD subjects. Herbert et al. (94), however, reported increased leftward planum temporale asymmetry in boys with autism. Gage et al. (93) showed rightward asymmetries for both planum temporale and posterior superior temporal gyrus. Such variability may be due to the homogeneity of ASD and differences in sample characteristics and/or methods. The present results show larger gray matter volumes for both left and right superior temporal gyri in the ASD group, relative to controls. In addition, the left superior temporal gyri of subjects with ASD showed no indication of being larger than their right hemisphere homolog, on average, which is consistent with the aforementioned studies. Either or both of these current findings have the potential to underlie sensory abnormalities.

While asymmetries in absolute volume were not observed in the current study, a rightward asymmetry in structural covariance was noted. That is, the average of the structural correlation coefficients between hemispheres was appreciably higher in the right hemisphere. Thus, there appears to be a significant rightward asymmetry of structural covariance of sensory-related cortical structures in the current sample. This structural covariance asymmetry could be suggestive of hyperconnectivity of sensory cortices within the right hemisphere. These findings are consistent with results from a number of recent studies using functional MRI to evaluate the resting-state functional connectivity in the brains of participants with ASD (76, 80). Both of these studies noted a pattern of hyperconnectivity in the right hemisphere of these subjects vs. controls.

While no study to our knowledge has reported such a structural covariance finding in the past, there are several potential interpretations grounded in the literature that are consistent with the current hypothesis. For instance, temporal cortices of the right hemisphere have been implicated in paralinguistic and pragmatic language processing, with left hemisphere counterparts playing an important role in linguistic (e.g., syntax and semantics) portions of language. Paralinguistic elements of language are important for understanding of communicative intent, beyond the literal meaning of words and sentences. These factors might include sarcasm, emotional content, metaphors, double meanings, other non-literal language, and prosody. The integration of both the linguistic and paralinguistic parts of language is essential for accurate discourse comprehension, and, thus, to successful social functioning. Previous studies have shown that patients with right hemisphere lesions made significantly more mistakes in discourse comprehension. These errors were specifically attributable to incorrect inferences about what was being said or read, due to patients’ interpretations being overly literal, which is also a common phenotype in the ASD population [see Mitchell and Crow (95), for a review]. Thus, rightward asymmetry of structural covariance may represent a dysfunction of connectivity between regions that play a role in paralinguistic processing.

### Limitations and Future Directions

While the current study may provide results that are consistent with our hypothesis and previous reports of individuals with ASD, there are several limitations that we should note. First, structural covariance and functional connectivity measures are only indirectly related to each other (96). Additionally, structural connectivity is more directly measured using diffusion tensor imaging (DTI). Furthermore, volume is only one aspect of the structures investigated in the current study. Other characteristics, such as thickness, curvature, and surface area could also be assessed in future studies, since they may represent different properties of cellular organization and/or development (97–99). Therefore, the structural covariation results presented here may not have direct functional/structural connectivity implications. Future studies should endeavor to characterize the link between structural covariance and functional/structural connectivity in autism. Such a characterization could be useful clinically, as structural MRI is in many ways more conveniently collected and analyzed than fMRI or DTI, particularly with lower functioning individuals with ASD. Covariance features extracted from automated structural MRI analyses could be amenable for use with infants and young children, and other patients who otherwise could not participate in fMRI recordings.

Another weakness of the current investigation is that the data analyzed here were not specifically collected to examine sensory dysfunction in ASD. While this fact should not change the structural covariance results, it means that no behavioral data related to sensory functioning were collected alongside anatomical data. Thus, we were unable to explore any potential links between anatomical and behavioral phenomena. Several previous studies have presented data examining relationships between structural covariance and behavior. For instance, one group has argued that various characteristics of structural covariance are useful as diagnostic predictors of patients with ASD (48). In addition, since correlation coefficients are a composite measure, we did not have values representing the strength of structural covariance for each participant. This statistical reality meant that we were unable to correlate other factors, such as age, with correlation results. Thus, future studies should continue this line of research in order to determine the association between structural covariance, behavior, and demographics, and the clinical usefulness of these neuroimaging and analysis techniques.

## Ethics Statement

This study was carried out in accordance with the recommendations of the Belmont Report as reviewed by the Colorado Multiple Institutional Review Board with written informed consent from all subjects or their guardians. Additionally, all children aged seven and above provided written assent prior to participating in the study. All subjects gave written informed consent/assent in accordance with the Declaration of Helsinki. The protocol was approved by the Colorado Multiple Institutional Review Board.

## Author Contributions

GC and DR contributed equally to the conceptualization, hypothesis development, data analysis and interpretation, writing, and editing of the current manuscript. SH and DR worked to recruit participants and acquire behavioral and MRI data with each subject. SH also assisted in the writing and editing of the article.

## Conflict of Interest Statement

The authors declare that the research was conducted in the absence of any commercial or financial relationships that could be construed as a potential conflict of interest.
