# Supplementary material for: Structural Covariance of Sensory Networks, the Cerebellum, and Amygdala in Autism Spectrum Disorder
Source: Front Neurol. 2017 Nov 27;8:615. doi: 10.3389/fneur.2017.00615 (PMC5712069; doi:10.3389/fneur.2017.00615)
Supplement: Supplementary file 1 [file Data_Sheet_1.pdf]

**Supplementary Materials**

Supplementary Table 1: Significance, mean volume, and standard deviation values for structural volumes that differed significantly between the ASD and TD groups via general linear model, controlling for intra-cranial volume and gender.

| Structures | F; P-value | FDR corrected P-value (q) | ASD Mean Volume (SD) $cm^3$ | TD Mean Volume (SD) $cm^3$ |
|---|---|---|---|---|
| LH Transverse Temporal Gyrus | 13.73; 0.00 | 0.00 | 1432.31 (300.95) | 1209.97 (156.17) |
| RH Transverse Temporal Gyrus | 10.99; 0.00 | 0.03 | 1131.94 (305.67) | 927.78 (190.71) |
| RH Banks of the STS | 10.91; 0.00 | 0.03 | 2282.69 (597.34) | 2682.66 (496.78) |
| Left Nucleus Accumbens | 7.77; 0.01 | 0.08 | 636.23 (196.03) | 748.11 (144.59) |
| Right Nucleus Accumbens | 16.12; 0.00 | 0.00 | 684.93 (156.30) | 835.65 (176.11) |
